# Industrial chemistry and chemoecology are linked together to realize a modern and sustainable chemistry

**DOI:** 10.1186/1752-153X-1-9

**Published:** 2007-03-20

**Authors:** Peter Saling

**Affiliations:** 1 BASF Aktiengesellschaft, Ludwigshafen, Germany, GUP/CE – Z 570, 67056 Ludwigshafen

## Abstract

Meeting society's needs without damaging the environment requires new ways of thinking. The link of industrial chemistry and chemoecology will be one of the key factors of sustainable development within the chemicals industry.

Industrial chemistry plays an important role in our society. Thousands of products and applications have increased the standard of living of billions of people in the last century. Life today without the benefits of industrial chemistry and the chemical industry would be difficult and of low quality [1]. Nevertheless, society is often critical of industrial chemistry and does not accept all products offered by it. Therefore it is an important obligation of manufacturers and scientists to convince people that chemicals can offer solutions to current problems. Changing of resources, of methodologies, of chemical individuals, technologies etc. in a competing economy with different types of industries requires an ongoing dialog and a realistic proof-of-concept to achieve the "license-to-operate" for the chemical industry. In this situation, a well-acknowledged journal with high-quality articles can bring essential contributions to current discussions with a high impact. The *Chemistry Central Journal *intends to be a platform for the discussion of new trends, processes and applications of chemicals. Chemoecology, is an important field for the evaluation of new and important impacts. It can help to show, in a scientific manner, where the weaknesses and strengths of different systems lie. Quantifying the sustainability benefits of industrial chemistry is therefore an important issue for the further development of this field of opportunities in the future. A realistic and validated estimate of innovative potentials of industrial chemistry by using quantitative methods is essential for the development of new products and processes. New approaches and developments of "Green Chemistry", "Green Engineering", "Chemoecology" etc. in combination with the development of industrial processes should be published in the *Chemistry Central Journal*.

Using life-cycle management tools and assessments of the entire product life cycle – from concept development to design and implementation, further to marketing, and finally, end-of-life issues – will be an important factor for successful new industrial processes and products. The chemical industry has played a key role in the development of such new tools and techniques as well as in impact assessment research, consistently allocating a large portion of its resources to research and development [2].

The design of new processes needs to consider aspects of the Chemoecology too. The link of industrial chemistry and Chemoecology will be one of the key factors of sustainable development within the chemicals industry.

Meeting society's needs without damaging the environment requires new ways of thinking. The chemical industry has a strong record of innovation – in products that meet customers' needs, in manufacturing processes that protect the environment and human health, and in solutions that directly address environmental problems [3].

To promote the communication and understanding between the academic community and the chemical industry is important. The best way to bridge the gap between the ideas that academia generates and the ideas that the product development industry seeks should be evaluated [4]. Communication on new topics within a worldwide network is crucial for additional increases in quality of life with chemicals for today and tomorrow. The *Chemistry Central Journal *will be a platform for the exchange of ideas in this exciting domain.
